# Extremely Rare Form of Impaction Bilateral Kissing Molars: Report of a Case and Review of the Literature

**DOI:** 10.1155/2016/2560792

**Published:** 2016-06-15

**Authors:** Tamer Zerener, Gurkan Rasit Bayar, Hasan Ayberk Altug, Serkan Kiran

**Affiliations:** Gulhane Military Medical Academy, Department of Oral and Maxillofacial Surgery, 06010 Ankara, Turkey

## Abstract

Kissing molars (KM) or rosette formation is a term that is used to describe impacted teeth contacting occlusal surfaces in a single follicular space and their roots pointing in opposite directions. In some cases kissing molars can be seen but occurrence of bilateral kissing molars is extremely rare phenomenon in the dental literature and the aetiology of this phenomenon is still unknown. In this paper we describe a case and review of the literature and discuss the management of this pathology. In our case, extremely rare form of impacted bilateral kissing molars was extracted surgically. The decision of extraction of asymptomatic kissing molars represents surgical dilemma. There may be many surgical complications; on the other hand in some cases surgical intervention is unavoidable. Few treatment options were described in the literature. This phenomenon can be sign of various medical conditions that may require further investigation. In this paper, our treatment option is in agreement with the literature suggesting the surgical removal of both teeth at either side of the mandible.

## 1. Introduction

The permanent teeth can be affected by eruption problems. The most affected ones are the mandibular and maxillary third molars, maxillary canines, central incisors, second mandibular and maxillary premolars, and rarely second molars (0.03–0.04% of all impacted teeth), respectively [1]. Kissing molars (KM) or rosette formation is a term that is used to describe impacted teeth contacting occlusal surfaces in a single follicular space and their roots pointing in opposite directions [2]. The condition of impaction type of teeth was described first by van Hoof in 1973 [3]. In some cases kissing molars can be seen but occurrence of bilateral kissing molars is extremely rare phenomenon in the dental literature and the aetiology of this phenomenon is still unknown [4, 5].

In this paper, we report a case with this extremely rare phenomenon and discuss the management of this phenomenon by reviewing the literature.

## 2. Case Report

**Figure 1:**
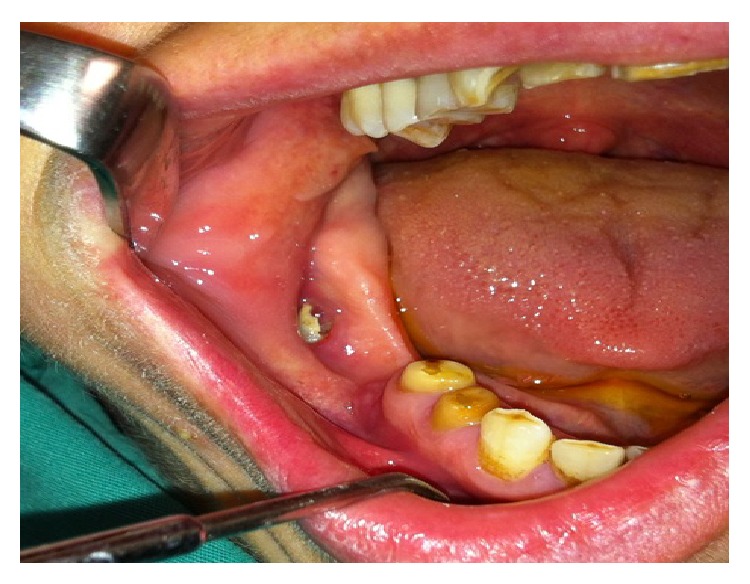
Intraoral view of the patient before the surgery.

**Figure 2:**
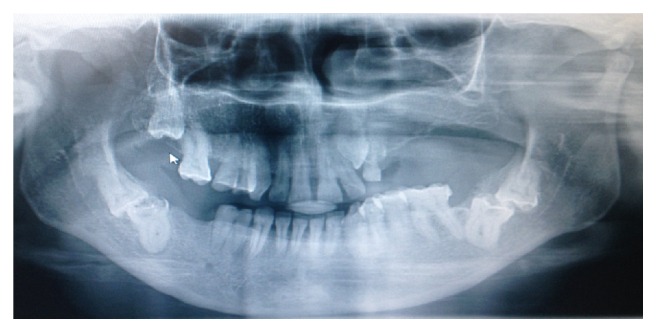
Orthopantomography showing the presence of kissing molars in each side of the mandible.

A 38-year-old female patient referred to the Department of Oral and Maxillofacial Surgery with a complaint of swelling at the right lower side of the angulus mandibula. Intraoral examination showed an expansion of buccal cortical plate and a palpable soft swelling over the residual alveolar ridge bone in the second and third molars region of lower mandible (Figure 1). Medically, the patient condition was fit and well, without any previously known allergic reaction, and she was not taking any medication. Orthopantomography revealed that there was bilateral impaction of the lower second and third molars in each side of the angulus region of the mandible. In each side of the mandible, the impacted teeth (the second and third molars) had their occlusal surfaces contacting each other in a single follicular space (Figure 2).

**Figure 3:**
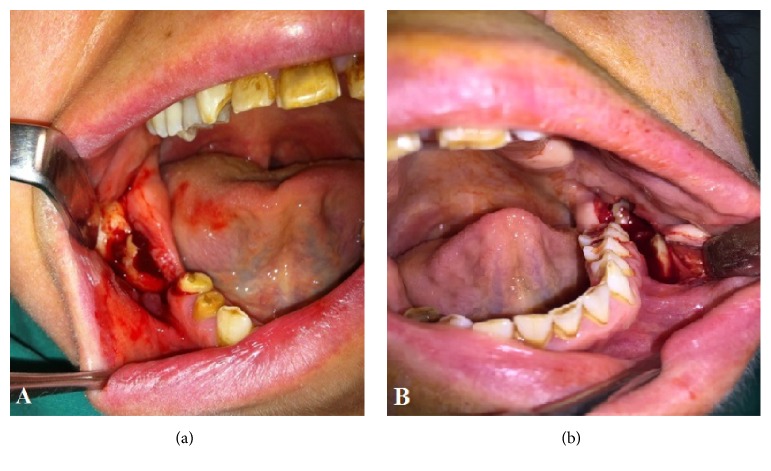
(a) Showing the surgical operation on the right side of the mandible and (b) on the left side of the mandible.

**Figure 4:**
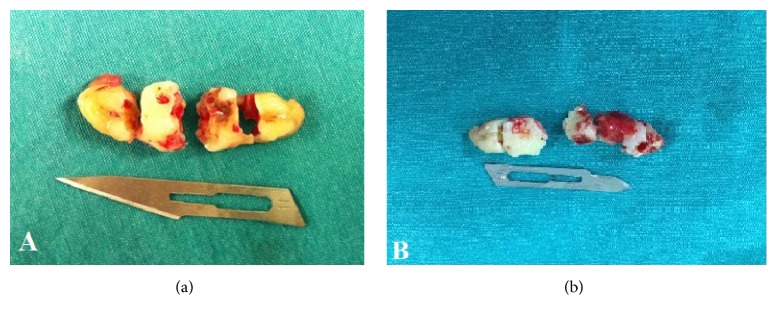
(a) Kissing molars extracted from the right side of the mandible and (b) from the left side of the mandible.

**Figure 5:**
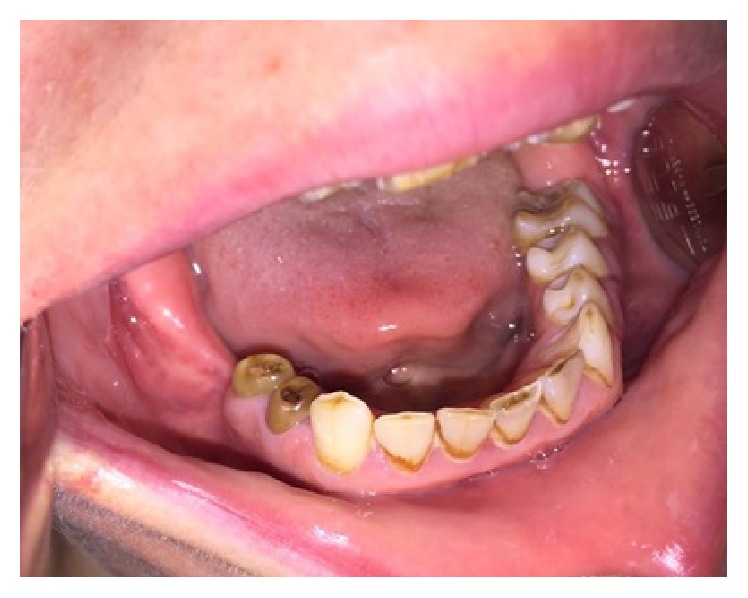
Intraoral view of the patient in recovery period.

**Figure 6:**
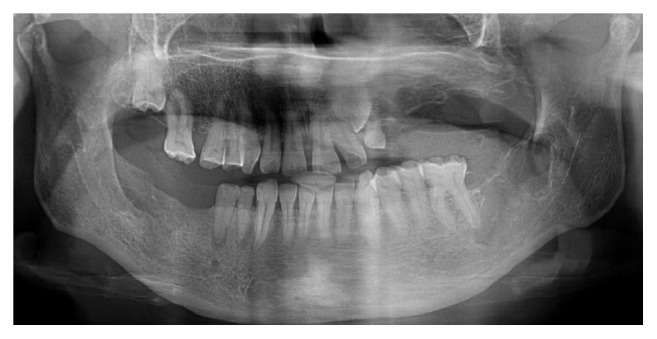
Panoramic view of the patient 4 months after the first surgery.

The stipulated treatment plan was the surgical removal of the kissing molars. Before the surgery a complete survey was performed, including blood tests. Results of the tests did not reveal any medical problem and/or risk condition. The surgical operation was performed under regional and infiltration anesthesia blocking the inferior alveolar, buccal, and lingual nerves. The patient was medicated preoperatively with 40 mg prednisolone for controlling the postoperative complications. The kissing molars were approached with the help of a vestibular incision. After the vestibular incision, mucoperiosteal flap was removed. Then, osteotomy was performed to expose the impacted kissing molars. The next step of the surgery was the sectioning of the third molar with surgical burs to minimize the quantity of bone removal and facilitate the extraction. After removal of the third molar, second molar was removed by the same approach. Following removal of kissing molars, the socket was rinsed with saline solution and residual follicular tissue was removed (Figures 3(a) and 4(a)). Finally, the operational region was sutured by interrupted stitches using 3/0 silk suture. After 3 months from the first operation, the same surgical intervention procedure was performed to the other side of the mandible (Figures 3(b) and 4(b)). Following the surgical operations, for controlling postoperative pain and infection, 1000 mg amoxicillin and 550 mg naproxen sodium were prescribed to the patient for a week. Swelling decreased gradually in the follow-up period after surgery. The sutures were removed seven days after the operations. The patient was kept on a soft diet for about two months. After that, recovery period was uneventful (Figures 5 and 6).

## 3. Discussion

Kissing molars are a form of impaction that is very rarely reported in dental literature. The term of kissing molars or rosette formation was first described in 1973. It refers to mandibular second and third molars which have occlusal surfaces containing each other in an enlarged single follicular space and roots pointing in opposite directions [3].

The distinction between unusual impaction and kissing molars or rosetting of molars is unclear and the aetiology of this phenomenon remains to be unknown [6, 7]. Multiple rosetting of molars or bilateral kissing molars had been associated with mucopolysaccharidosis (MPS) [8]. It is a group of inherited metabolic disorders and an enzyme abnormality accumulation of mucopolysaccharides in different tissues of the body [9]. It may be related to this phenomenon. However, it has not been cleared yet. Therefore, clinicians should be prompted to establish definitive rules regarding the relation between MPS and this kind of impaction conditions. Cawson [10] reported a patient affected with MPS and suggested that MPS could be a possible aetiological factor for occurrence of kissing molars. Also, Nakamura et al. [11] reported multiple rosetting of molars in their 2 out of 4 patients with MPS.

The decision for extraction of asymptomatic kissing molars represents surgical dilemma. Many complications could be seen after surgical intervention, such as mandibular fractures during the surgery or postoperatively, dry socket, damage to the inferior alveolar nerve (0.5 to 5%) and lingual nerve (0.2 to 2%), osteomyelitis, and temporomandibular joint (TMJ) disorders, especially internal derangements [12, 13].

On the other hand, maintenance of kissing molars may cause other complications, such as reduction of the bone of mandible increasing the risk of mandibular fracture, pericoronitis, local pain, cystic changes, and root resorption of adjacent teeth [12–14]. In order to prevent or reduce these kinds of complication, surgical planning is necessary. Panoramic radiography is considered as the gold standard in most cases. CT scans must be used for evaluation of proximity of inferior alveolar nerve channel. Utmost care must be taken to avoid lingual nerve injury.

In English dental literature kissing molars can be seen in unilateral or bilateral forms. Most of them are in unilateral form. We reviewed English dental literature regarding bilateral form of impaction of kissing molars and put them together in a table (Table 1) [3, 11, 15–18]. Symptoms, radiographic presentation, related medical problems, treatment alternatives, postoperative complications, and histopathological findings are summarized in this table.

## 4. Conclusion

In dental practice, clinicians encounter various types of impaction of teeth. Kissing molars is another impaction type of teeth. However, the phenomenon of this issue has not been well described yet. Few treatment options were described in the literature. This phenomenon can be sign of various medical conditions that may require further investigation. In this paper, our treatment option was in agreement with the literature suggesting the surgical removal of both teeth at either side of the mandible.

## Figures and Tables

**Table 1:** Cases reported as bilateral kissing molars in the English dental literature.

Author/year	Sex/age	Symptom	Radiographic presentation	Medical problems	Treatment	Postop	Histopathology
van Hoof 1973 [3]	Male 31	None	Bilateral Mandibular Impaction	Intellectually challenged	Maintained	—	—
Robinson et al. 1991 [15]	Male 25	None	Bilateral Mandibular Impaction	None	Maintained	—	—
Nakamura et al. 1992 [11]	Male 25	None	Bilateral rosette formation in both jaws	MPS	Maintained	—	—
Nakamura et al. 1992 [11]	Male 17	None	Bilateral rosette formation in both jaws	MPS	Maintained	—	—
Nakamura et al. 1992 [11]	Male 21	None	Bilateral rosette formation in both jaws	None	Maintained	—	—
Bakaeen and Baqain 2005 [16]	Male 23	Facial pain	Bilateral Mandibular Impaction	None	Surgical removal under GA	Trismus and dry socket	—
Sa Fartes et al. 2014 [17]	Male 33	None	Bilateral Mandibular Impaction	None	Surgical removal under LA	—	Dentigerous cyst
Kiran et al. 2014 [18]	Female 18	None	Bilateral Mandibular Impaction	None	Surgical removal under GA	—	—
Present case 2015	Female 38	Swelling over the right lower side of the face	Bilateral Mandibular Impaction	None	Surgical removal under LA	—	—

MPS: mucopolysaccharidosis, GA: general anesthesia, and LA: local anesthesia.
